# *In Vivo* Evaluation of a Self-Excitatory Near-Infrared ImmunoSCIFI Probe

**DOI:** 10.1021/acs.bioconjchem.5c00506

**Published:** 2026-01-07

**Authors:** Katie Gristwood, Saimir Luli, Helen J Blair, Kenneth S. Rankin, James C. Knight

**Affiliations:** 1 School of Natural and Environmental Sciences, 5994 Newcastle University, Newcastle upon Tyne NE1 7RU, U.K.; 2 Preclinical In Vivo Imaging, Translational and Clinical Research Institute, Newcastle University, Newcastle upon Tyne NE2 4HH, U.K.; 3 Translational and Clinical Research Institute, Newcastle University, Newcastle upon Tyne NE1 7RU, U.K.; 4 Wolfson Childhood Cancer Research Centre, Newcastle Upon Tyne NE1 7RY, U.K.; 5 North of England Bone and Soft Tissue Tumour Service, 5983 Newcastle Upon Tyne Hospitals NHS Foundation Trust, Freeman Road, Newcastle Upon Tyne NE7 7DN, U.K.; 6 Newcastle Centre for Cancer, 5994 Newcastle University, Newcastle Upon Tyne NE1 7RU, U.K.

## Abstract

Secondary Cerenkov-induced fluorescence imaging (SCIFI) utilizes blue-weighted Cerenkov luminescence from radioactive decay to excite proximal fluorophores that emit near-infrared light with optimal penetrance through biological tissues and offers potential utility in clinical imaging applications, including guidance of surgical resection. Recently, we developed a self-excitatory immunoSCIFI probe based on an antibody modified with the Cerenkov luminescence generating radioisotope zirconium-89 and a near-infrared boron-dipyrromethene dye (BOD665) and observed an immunoSCIFI signal in *in vitro* cell-based experiments. In this study, we have evaluated the *in vivo* application of immunoSCIFI using a clinically relevant orthotopic mouse model of dedifferentiated chondrosarcoma as a reproducible, high contrast setting in which to challenge the optical method under bone and soft tissue attenuation. Herein, we report the synthesis, characterization, preclinical imaging, and *ex vivo* biodistribution analysis of a novel immunoSCIFI probe, [^89^Zr]­Zr-DFO-MT1-MMP-BOD665, based on a murine monoclonal immunoglobulin G (IgG) with high binding specificity for the sarcoma biomarker MT1-MMP. Both *in vivo* imaging and *ex vivo* data indicated significantly higher total uptake and femur-to-muscle ratios in the inoculated femurs with high MT1-MMP expression relative to contralateral femurs. These preliminary findings establish that antibody-mediated SCIFI can operate *in vivo* with favorable signal-to-background performance under physiologically relevant photon attenuation. The study therefore provides a methodological foundation for future SCIFI probes, for which rigorous specificity testing and broader biomarker panels will be pursued separately.

## Graphical abstract

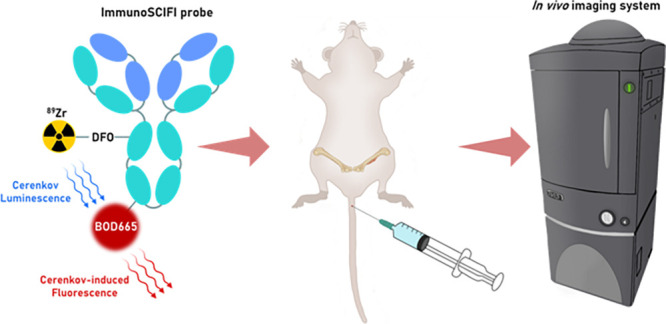

## Introduction

Secondary Cerenkov-induced Fluorescence Imaging (SCIFI) is an emergent biomedical optical imaging technique that utilizes the faint blue-weighted Cerenkov luminescence (CL) produced during radioactive decay to excite near-infrared (NIR) fluorophores in close proximity, leading to emission of NIR light (650–1350 nm) with optimal penetrance through biological tissues.^1^ This *in situ* excitation method contrasts with traditional external excitation techniques that are hindered by scattering and attenuation of the incident light.^2^

The conceptual foundations of SCIFI evolved through several key studies. Liu et al. first demonstrated “radiation-luminescence excitation” of quantum dots (QD655) when coadministered with [^131^I]­NaI in athymic nude mice.^3^ Shortly thereafter, Dothager et al. described the phenomenon as “Cerenkov radiation energy transfer (CRET)” when exciting Qtracker705 using CL from ^18^F and ^64^Cu.^4^ The term SCIFI was subsequently introduced by Thorek et al., who reported NIR-shifted emissions upon colocalization of QD605 with [^89^Zr]­Zr-DFO-trastuzumab in mice bearing HER2/neu-positive xenograft tumors.^5^ Together, these studies provided early evidence for the utility of CL-mediated excitation in biomedical optical imaging applications, including *in vivo* detection of molecular biomarkers of disease.

Previously, we confirmed the suitability of boron-dipyrromethene (BODIPY) dyes in combination with ^89^Zr for SCIFI applications based on the rapid acquisition (<5 min) of CL-induced emission spectra and high photostability, despite prolonged exposure to ionizing radiation.^6^ We subsequently reported the synthesis and *in vitro* evaluation of [^89^Zr]­Zr-DFO-trastuzumab-BOD665; a novel antibody-based self-excitatory immunoSCIFI probe that offered high binding specificity for the cancer biomarker HER2 and near-infrared fluorescence emissions induced by ^89^Zr-generated CL. This study also included fluorescence and CT imaging experiments based on hemoglobin-infused agarose phantoms that showed a 2.05-fold enhancement in-depth penetration of SCIFI signal upon combination of ^89^Zr and BOD665, relative to ^89^Zr-generated CL.^7^

Building upon these preliminary findings, we have advanced the preclinical development of immunoSCIFI and herein report a study primarily designed to evaluate its applicability *in vivo* (Figure 1). To this end, an established orthotopic murine model of dedifferentiated chondrosarcoma produced by intrafemoral inoculation of HT1080 cells that overexpress matrix metalloproteinase-1 protein (MT1-MMP, also termed MMP14)^8^ provided a robust, high contrast setting for imaging validation, while in-depth analyses of target specificity are planned for future studies. A novel anti-MT1-MMP immunoSCIFI probe was designed for this study based on a murine monoclonal IgG (MSX LEM-2/15.8, Millipore) that we have previously modified with ^89^Zr and IRDye800CW via a site-specific chemoenzymatic approach for dual-modal (PET/NIRF) imaging of MT1-MMP overexpression in the same mouse model.^8^ In the present study, the antibody is modified in a stochastic manner with the CL-generating isotope ^89^Zr and the CL-activatable fluorophore BOD665.

**Figure 1:**
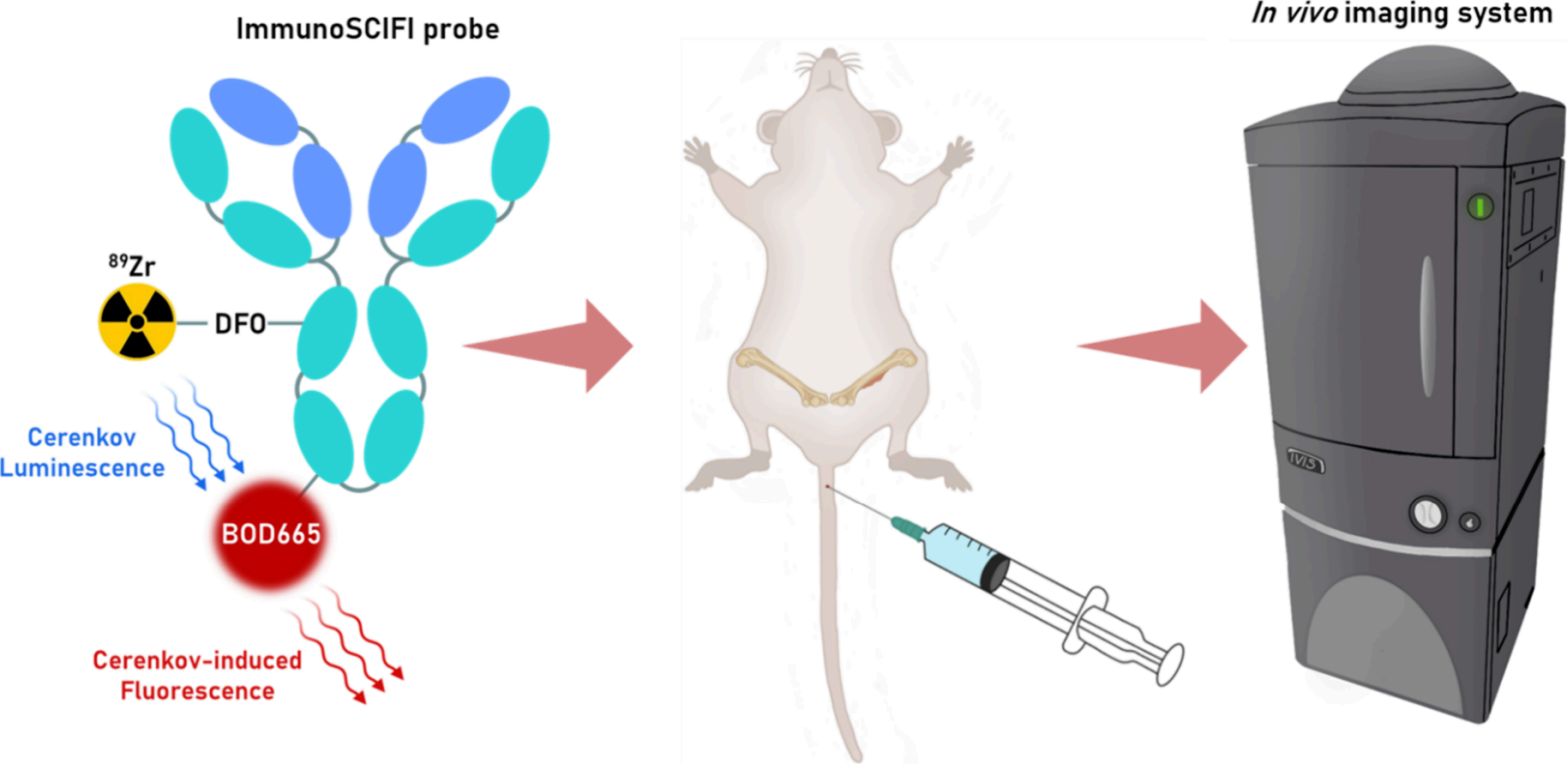
Photonic energy transfer from ^89^Zr-generated Cerenkov luminescence to BOD665 within the [^89^Zr]­Zr-DFO-anti-MT1-MMP-BOD665 immunoSCIFI probe yielding near-infrared shifted SCIFI emissions. Intravenous injection of the probe into mice bearing femoral HT1080 tumors enables secondary Cerenkov-induced fluorescence imaging *in vivo*.

Here, we describe the synthesis and physicochemical characterization of the self-excitatory immunoSCIFI agent [^89^Zr]­Zr-DFO-MT1-MMP-BOD665 and present its first *in vivo* assessment—conducted by *in vivo* imaging and corroborative *ex vivo* biodistribution analyses in MT1-MMP-positive HT1080 tumor-bearing mice—to provide proof-of-concept data for applying immunoSCIFI under physiological conditions.

## Results & Discussion

### Synthesis and Characterization of DFO-anti-MT1-MMP-BOD665

Anti-MT1-MMP immunoglobulin (IgG) was first modified with BODIPY 650/665-X-NHS ester (BOD665) through stochastic modification of endogenous lysine residues via amide bond formation at ε-amino positions, with an isolated yield of 53% after purification and degree-of-labeling of 2, determined by UV–vis absorption measurements (Figure S2 and Table S1). After conjugation the absorption maximum (*A*_max_) of BOD665 increased from 655 to 665 nm, consistent with previously reported antibody-fluorophore conjugates.^9^ While CL is strongest in the UV-blue region, its broad emission spectrum provides sufficient overlap with the excitation profile of the BODIPY dye to enable SCIFI, while the resulting near-infrared emission enhances tissue penetration *in vivo*. The bifunctional ^89^Zr chelator p-SCN-Bn-deferoxamine (p-SCN-Bn-DFO) was then conjugated to anti-MT1-MMP-BOD665 through lysine-directed thiourea formation, resulting in DFO-anti-MT1-MMP-BOD665 with an isolated yield of 99% after purification (Figure 2A). Based on our previous findings with related immunoconjugates, the concurrent bioconjugation of BODIPY and DFO applied in this work is unlikely to compromise binding.^7,8^

**Figure 2:**
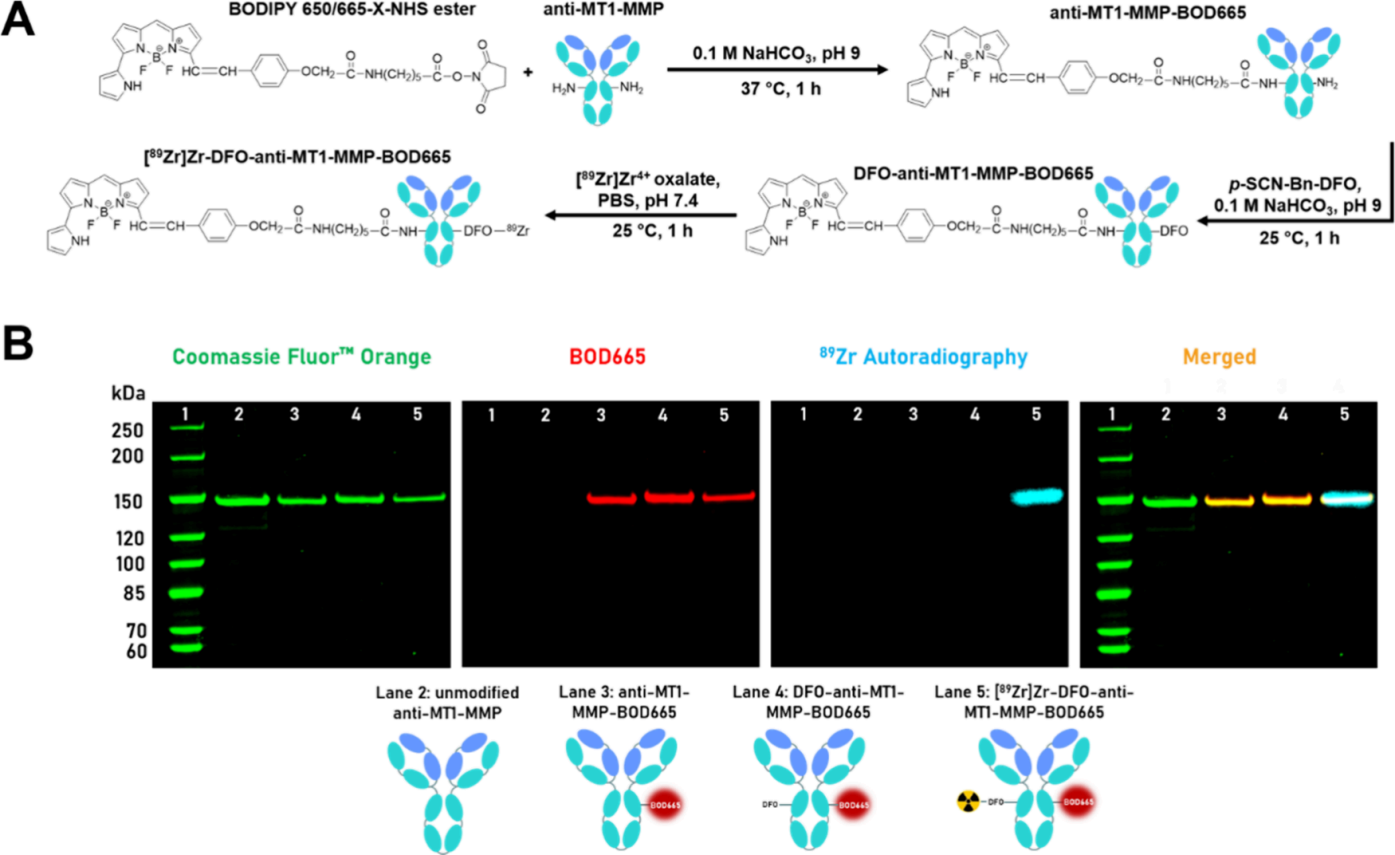
Synthesis and characterization of the immunoSCIFI probe. (A) Reaction scheme for the synthesis of radioimmunoconjugate [^89^Zr]­Zr-DFO-anti-MT1-MMP-BOD665. (B) Radio-SDS-PAGE of unmodified anti-MT1-MMP antibody (lane 2), anti-MT1-MMP-BOD665 (lane 3), DFO-anti-MT1-MMP-BOD665 (lane 4), and [^89^Zr]­Zr-DFO-anti-MT1-MMP-BOD665 (lane 5) compared to a protein ladder.

Sodium dodecyl sulfate polyacrylamide gel electrophoresis (SDS-PAGE) analysis revealed colocalization of the BOD665 and anti-MT1-MMP bands at ∼150 kDa, indicating the conjugation of the dye to the antibody (Figure 2B).

### Radiolabeling and Characterization

The DFO-anti-MT1-MMP-BOD665 conjugate was labeled with neutralized ^89^Zr at a specific activity of 0.1 MBq/μg with a radiolabeling efficiency of >99%, determined by radio instant thin-layer chromatography (radio-iTLC; Figure S3) and used without further purification. SDS-PAGE analysis and subsequent imaging showed the colocalization of the radionuclide with the anti-MT1-MMP protein and BOD665 fluorescence signals (Figure 2B), confirming the synthesis of the [^89^Zr]­Zr-DFO-anti-MT1-MMP-BOD665 immunoSCIFI probe.

### *In Vivo* and *Ex Vivo* Evaluation

[^89^Zr]­Zr-DFO-anti-MT1-MMP-BOD665 was administered intravenously into mice bearing HT1080 femoral tumors; a recently developed clinically relevant murine model of MT1-MMP positive dedifferentiated chondrosarcoma.^8^ Mice were imaged for ^89^Zr-generated Cerenkov luminescence (CL; ex. block, em. 500 nm) and SCIFI signal (ex. block, em. open filter) at 24, 48, and 72 h p.i. As previous IVIS imaging of ^89^Zr-generated CL revealed the greatest emissions within the 500 nm emission filter (Figure S1),^6^ the absence of emissions from the probe within this filter indicates efficient SCIFI energy transfer to the BOD665 fluorophore. Emissions were detected within the region of the HT1080 inoculated femurs when the live mice were imaged in open filter settings, whereas there was an absence of signal from the contralateral femurs (Figure 3A). Taken together, these findings are consistent with MT1-MMP binding by the modified IgG and with CL-mediated excitation of the BOD665 fluorophore, although definitive confirmation will require additional specificity-orientated mechanistic studies.

**Figure 3:**
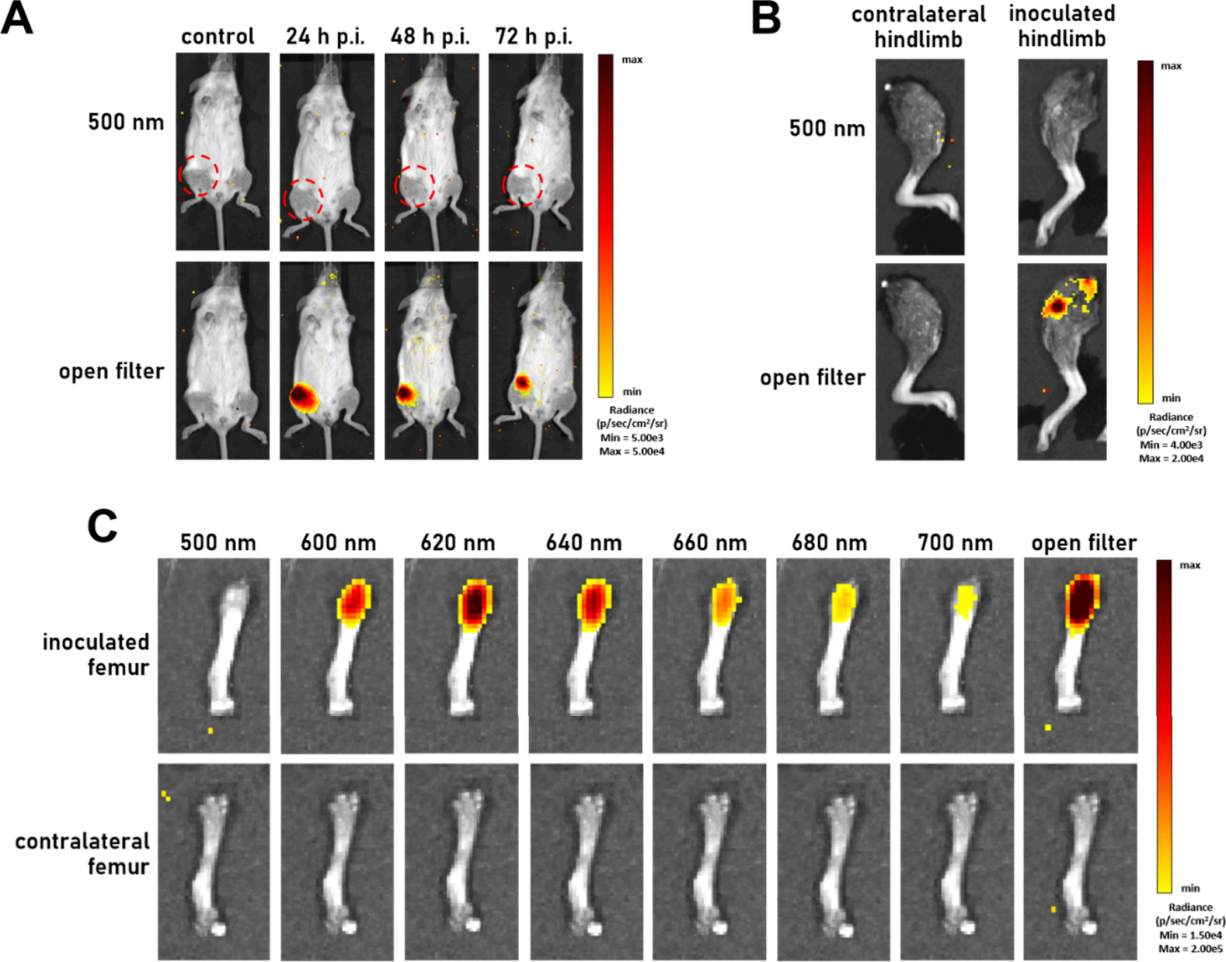
Representative IVIS images showing (A) mouse bearing HT1080 (high MT1-MMP) sarcoma tumors (right femur in the supine position; red dashed circle) acquired at 24, 48, and 72 h p.i. of [^89^Zr]­Zr-DFO-anti-MT1-MMP-BOD665. Control mice bore femoral HT1080 tumors but did not receive the immunoSCIFI probe. Mice were imaged for Cerenkov luminescence (ex. block, em. 500 nm) and fluorescence (ex. block, em. open). (B) Involved and contralateral hindlimbs *ex vivo* imaged for Cerenkov luminescence and fluorescence. (C) Involved and contralateral femurs *ex vivo* imaged for Cerenkov luminescence and fluorescence (ex. block, em. 600–700 nm, and open filter).

Following the final imaging session, the hindlimbs were removed from the culled mice and imaged using the same settings as previously implemented. Again, the absence of CL was noted in both inoculated and contralateral hindlimbs at 500 nm, whereas prominent SCIFI emissions were detected along the axis of the femur using an open emission filter (Figure 3B).

The femurs (Figure 3C) and surrounding muscle tissue (Figure S3) were then excised from the hindlimbs and imaged for CL (500 nm) and SCIFI (open filter) and six additional emission filters (600–700 nm, 20 nm increments). The peak of the SCIFI emissions was located within the 620 nm emission filter (bandwidth 10 nm: 610–630 nm range) before monotonically decreasing toward 700 nm in the inoculated femurs. The absence of CL or SCIFI emissions from the contralateral femurs was also noted (Figure 3C). These findings are consistent with *in vivo* images taken of the mice across a similar range of emission filters (Figure S2). An apparent shift in the emission maximum of BOD665 was observed, although this can be attributed to the greater complexity of an *in vivo* environment compared to *in vitro* conditions. For example, the concentration of the fluorophore in *in vivo* settings is typically lower than in *in vitro* environments, an important distinction as dye concentration is known to cause spectral variation.^10^ Furthermore, hemoglobin contributes to both attenuation and spectral distortion of fluorescence signals, particularly in the near-infrared region.^11^ The intraosseous location of the tumors provides an analogous model for sarcomas diagnosed in patients,^8^ however the presence of the bone could further distort the fluorescence spectra due to the heterogeneity in optical properties compared to surrounding soft tissues.^12^

Biodistribution analysis of the immunoSCIFI probe was performed by gamma counting, expressed as a percentage of injected ^89^Zr activity per gram of tissue (%IA/g), across major tissues (Table 1). The overall biodistribution profile is consistent with similar intact IgG-based radioimmunoconjugates at 72 h p.i.^13^ Statistical analysis revealed significantly higher uptake of [^89^Zr]­Zr-DFO-anti-MT1-MMP-BOD665 in the MT1-MMP-overexpressing femurs compared to the MT1-MMP-low femurs (Figure 4A). Likewise, the femur-to-muscle ratio was significantly greater for the involved femurs (Figure 4B). Furthermore, the SCIFI signal measured during *ex vivo* imaging of the femurs was quantified through region of interest (ROI) analysis and corroborated the findings from the gamma counter analysis (Figure 4C,D). For the contralateral femur, the femur-to-muscle ratio determined by fluorescence emission data is notably lower than the corresponding value ascertained by gamma counting, likely due to instrument-specific limitations in sensitivity and optical attenuation. Similarly, the disparity between liver uptake and optical signal reflects the well-characterized poor recoverability of NIR emissions from deep visceral organs, in contract to the more favorable intraosseous tumor environment.

**Table 1:** *Ex Vivo* Biodistribution Data (%IA/g ± SD) for [^89^Zr]­Zr-DFO-anti-MT1-MMP-BOD665 in Mice Bearing HT1080 (High MT1-MMP) Tumors (*n* = 4) at 72 h p.i.

tissue	**[** **^89^** **Zr]Zr-DFO-anti-MT1-MMP-BOD665 uptake (%IA/g)**
blood	10.63 ± 1.08
inoculated femur	11.74 ± 2.63
proximal thigh muscle	1.46 ± 0.14
contralateral femur	6.44 ± 2.35
contralateral thigh muscle	1.07 ± 0.15
heart	3.63 ± 2.10
lung	4.25 ± 1.02
liver	11.88 ± 3.34
spleen	5.01 ± 1.39
stomach	0.44 ± 0.08
small intestine	0.80 ± 0.06
large intestine	1.27 ± 0.27
pancreas	1.65 ± 0.95
kidneys	3.99 ± 0.48
skin	1.08 ± 0.41
adipose	1.14 ± 0.54

**Figure 4:**
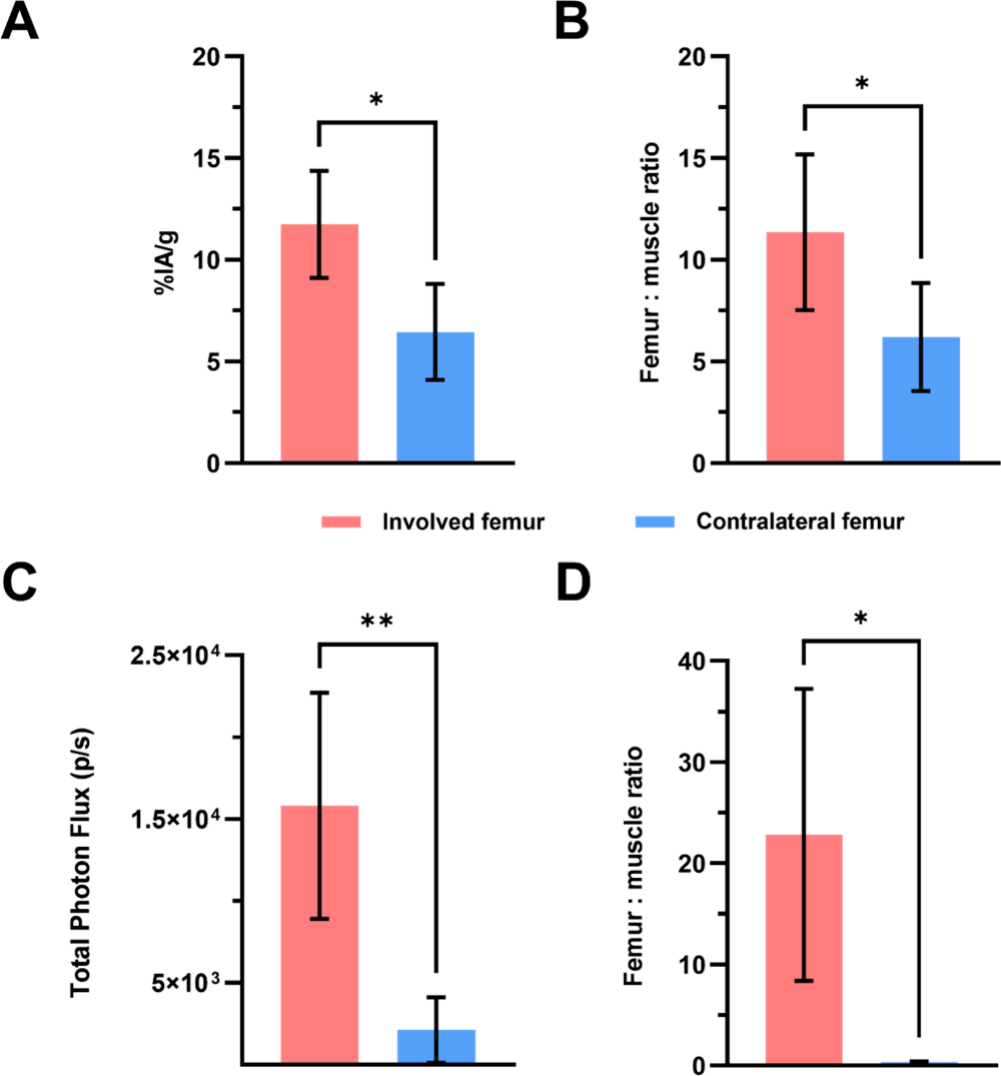
Fluorescence and radioactive emission data collected 72 h p.i. of [^89^Zr]­Zr-DFO-anti-MT1-MMP-BOD665 in mice bearing HT1080 (high MT1-MMP) sarcoma femoral tumors. (A) Percentage of the injected ^89^Zr activity per gram (%IA/g) retained in the involved and contralateral femurs (*n* = 4). (B) Femur-to-muscle ratio of ^89^Zr %IA/g in involved and contralateral femurs (*n* = 4). (C) Fluorescence emissions measured as the total photon flux (photons/second; p/s) at 620 nm in involved and contralateral femurs (*n* = 4). (D) Femur-to-muscle ratio of fluorescence emissions at 620 nm in involved and contralateral femurs (*n* = 4). Significance is indicated as follows: *P < 0.05 and **P < 0.01. Error bars represent standard deviation (SD).

Although intact IgG constructs exhibit a baseline degree of tumor localization via the enhanced permeability and retention (EPR) effect, the uptake of [^89^Zr]­Zr-DFO-anti-MT1-MMP-BOD665 in MT1-MMP-overexpressing femurs exceeds values commonly reported for nontargeted IgGs at comparable time points. In addition, a closely related dual-modal analogue of this antibody clone modified with [^89^Zr]­Zr-DFO and a NIR dye retained >80% immunoreactivity and demonstrated blocking sensitive uptake in the same HT1080 model.^8^ Taken together, these observations provide compelling–though still yet inferential–evidence that the immunoSCIFI signal in the orthotopic tumors is consistent with MT1-MMP engagement. Comprehensive biological validation and definitive confirmation of *in vivo* specificity will be pursued in a forthcoming, fully powered study combining excess-antibody blockade and imaging in MT1-MMP deficient tumors, thereby complementing the methodological focus of the present work.

## Conclusions

In this work we synthesized a dual-labeled, self-exciting antibody probe, [^89^Zr]­Zr-DFO-anti-MT1-MMP-BOD665, and provided the first demonstration of antibody-mediated SCIFI under *in vivo* conditions using a clinically relevant murine sarcoma model. Photon transfer from ^89^Zr-generated CL to the BOD665 fluorophore produced a robust near-infrared signal that was detectable through bone and soft tissue without conventional sources of external excitation. The uptake pattern observed in orthotopic tumors is consistent with MT1-MMP engagement, but the primary contribution of this study is the methodological validation of immunoSCIFI as an *in vivo* optical imaging modality. These findings establish a platform for future investigations aimed at comprehensive target-specific confirmation and at extending the technique to additional biomarkers and disease settings.

## Experimental Procedures

### General Methods

Unless noted otherwise, reagents and consumables were purchased from Fisher Scientific and used as received. Ultrapure water was produced with a Select Fusion system (SUEZ) to a resistivity of ≥ 18.2 MΩ·cm (25 °C). UV–vis absorbance readings were collected on a NanoDrop OneC microvolume spectrophotometer (NanoDrop Technologies). Radioactivity measurements were performed using either a CRC-25 dose calibrator (Capintec) or a Wizard 2480 gamma counter (PerkinElmer). Radiolabel incorporation into immunoconjugates was assessed by instant thin-layer chromatography (iTLC) on iTLC-SA glass microfiber strips (Agilent). Developed strips were visualized by autoradiography on an Amersham Typhoon imager and quantified in ImageQuant (both GE Healthcare).

### Preparation of Anti-MT1-MMP-BOD665

Four 100 μg portions of anti-MT1-MMP antibody (MSX LEM-2/15.8, Millipore; ∼100 μL each) were pooled and diluted with 0.1 M NaHCO_3_ (pH 8.3; 100 μL). The antibody solution was buffer-exchanged by centrifugal filtration using prerinsed 30 kDa MWCO, 0.5 mL spin filters (Amicon) for three cycles (12,000 × g, 8 min per cycle). After each centrifugation, 0.1 M NaHCO_3_ (pH 8.3) was added to restore the retentate to 500 μL. The antibody was recovered by inverting the filter devices and centrifuging (2,500 × g, 3 min), then adjusted to 2 mg/mL with 0.1 M NaHCO_3_ (pH 8.3) based on UV–vis absorbance. BODIPY 650/665-X NHS ester (1 mg) was dissolved in DMSO to prepare a 3 mg/mL stock solution, with brief sonication (5 min) to aid dissolution. A 50-fold molar excess of dye (from the DMSO stock) was then added to the antibody solution (285 μg in 200 μL; final DMSO 10% v/v), and the reaction mixture was incubated at 25 °C for 1 h with shaking (450 rpm).

### Size Exclusion Chromatography

Crude anti-MT1-MMP–BOD665 conjugates were purified by size exclusion chromatography using Sephadex G-50 resin (Sigma-Aldrich) packed into a 2 mL glass mini-column. Elution was performed with sterile PBS (pH 7.2; Gibco), collecting 100 μL fractions. The immunoconjugate typically eluted in fractions 8–12, while unreacted dye was retained on the column. Collected fractions were screened by UV–vis absorbance at 280 and 665 nm (λ_max_ for BOD665), and the conjugate-containing fractions were pooled and concentrated using a single centrifugal filtration step as described above.

### Degree-of-Labeling Determination

The mean number of BOD665 dyes conjugated to each antibody (DOL_BOD665_) in the purified immunoconjugate solution was calculated based on UV–vis absorption spectroscopy data (Figure S2 and Table S1) using the following equations:

$${\mathrm{DOL}}_{\mathrm{BOD}665}=\frac{A_{665}\times \mathrm{MW}[\mathrm{Ab}]}{\mathrm{Abconc}.\left(\frac{\mathrm{mg}}{\mathrm{mL}}\right)\times \varepsilon [\mathrm{BOD}665]}$$

where

$$\mathrm{Antibody}\;\mathrm{concentration}\;(\mathrm{mg}/\mathrm{mL})=10\times \frac{A_{280}-(A_{665}\times \mathrm{CF})}{\varepsilon_{1}\%[\mathrm{Ab}]}$$

CF = correction factor (*A*_280_/*A*_665_), ε_1_% = percent molar attenuation coefficient for a 10 mg/mL IgG solution.

The molar attenuation coefficient (ε) of BOD665 was determined from a standard curve via UV–vis absorption spectroscopy, for both unconjugated (ε_655_ = 84,590 M^–1^ cm^–1^) and conjugated (ε_665_ = 65,210 M^–1^ cm^–1^) states of the dye.

### DFO Attachment

A stock solution of p-SCN-Bn-DFO (Macrocyclics; 1-(4-isothiocyanatophenyl)-3-[6,17-dihydroxy-7,10,18,21-tetraoxo-27-(N-acetylhydroxylamino)-6,11,17,22-tetraazaheptaeicosine] thiourea) was prepared in DMSO at 2 mg/mL. A 15-fold molar excess of p-SCN-Bn-DFO (from the stock) was added to anti-MT1-MMP–BOD665 (1.5 mg/mL, 100 μL) in 0.1 M NaHCO_3_ buffer (pH 8.9–9.0). The reaction vessel was wrapped in aluminum foil to minimize light exposure and incubated at 37 °C for 1 h with shaking (450 rpm). The resulting DFO-anti-MT1-MMP-BOD665 conjugate was purified by centrifugal filtration as described above, with buffer exchange into PBS (pH 7.2).

### Radiolabeling

[^89^Zr]Zr in 1 M oxalic acid (PerkinElmer) was adjusted to approximately neutral pH by stepwise addition of 1 M Na_2_CO_3_. The pH was checked using narrow-range pH indicator paper 2–3 min after Na_2_CO_3_ addition. An aliquot of the neutralized ^89^Zr solution (∼13 MBq) was combined with DFO-anti-MT1-MMP-BOD665 (130 μg) to provide a target-specific activity of 0.1 MBq/μg, and the mixture was incubated at 25 °C for 1 h with shaking (450 rpm). Radiolabeling yield and radiochemical purity were assessed by radio-iTLC using 50 mM EDTA (pH 5.5) as the mobile phase. Under these conditions, the radioimmunoconjugate remained at the origin (*R*_f_ = 0), whereas unchelated ^89^Zr migrated with the solvent front (*R*_f_ = 0.8–1.0).^14^ {Chin, 2012 #14}­{Vosjan, 2010 #1}­{Vosjan, 2010 #1}­{Vosjan, 2010 #3}­{Vosjan, 2010 #1}­{Vosjan, 2010 #1}

### Radio-SDS-PAGE

[^89^Zr]­Zr-DFO-anti-MT1-MMP-BOD665 was characterized by SDS-PAGE. Antibody samples (≤7.5 μL, volume adjusted according to concentration) were combined with NuPAGE 4X LDS sample buffer (2.5 μL) and deionized water (≤6.5 μL, volume adjusted as required), to give a final volume of 10 μL. Samples were protected from light (foil-wrapped) and heated at 70 °C for 10 min with shaking (450 rpm). Protein standards (ThermoScientific PageRuler Unstained High Range Protein Ladder) and prepared samples were loaded onto NuPAGE 3–8% Tris-Acetate, 10-well mini-gels (1.0–1.5 mm) and electrophoresed for 1 h at 150 V using NuPAGE 1X Tris-Acetate SDS Running Buffer. Following electrophoresis, gels were rinsed in deionized water (∼200 mL; 3 × 5 min) and stained with Coomassie Fluor Orange (50 mL) for 1 h. Excess stain was removed by a brief wash in 1 M acetic acid (≤1 min) in, followed by a final rinse in deionized water prior to imaging. Fluorescence scanning was performed on an Amersham Typhoon Bioimager (GE) to detect Coomassie Fluor Orange (Cy2: λ_ex._ = 488 nm, λ_em._ = 525 nm) and BOD665 (Cy5: λ_ex_. = 635 nm, λ_em._ = 670 nm), after which the gel was imaged by digital ^89^Zr autoradiography.

### Cell Culture

HT1080 cells (high MT1-MMP) were obtained from the American Type Culture Collection (ATCC) and cultured in RPMI-1640 medium (Sigma-Aldrich) supplemented with 10% fetal bovine serum (FBS, Gibco), 200 mM l-glutamine, 10,000 units penicillin, and 10 mg/mL streptomycin (Sigma-Aldrich), and maintained in a humidified 5% CO_2_ environment at 37 °C. Cells were harvested at 80–90% confluency and passaged using 0.25% trypsin-EDTA solution (Sigma-Aldrich) with a subcultivation ratio of 1:15 every 3–4 days. The cumulative length of the culture was <6 months following retrieval of cells from liquid nitrogen storage. Cells were tested regularly for the absence of mycoplasma.

### *In Vivo* Studies

#### Ethics approval

All animal procedures were reviewed and approved by the Newcastle University Animal Welfare and Ethical Review Body (AWERB) and were performed in compliance with the UK Animals (Scientific Procedures) Act 1986 under UK Home Office Project License P74687DB5. All work involving handling and administration of ^89^Zr-labeled materials was conducted in accordance with institutional radiation protection procedures and local rules. Administered activities reflect typical values used in preclinical antibody-based PET imaging. Female NOD SCID gamma (NSG; Cg.-PrkdscidIl2rg tm1Wjl/SzJ) mice from an in-house colony (females, n = 7, 15–17 weeks old at the time of probe administration) were housed in specific pathogen-free conditions in individually ventilated cages with sterile bedding, *ad libitum* water, and diet (irradiated no. 3 breeding diet, SDS) in a facility with a 10 h light-dark cycle, and controlled temperature and humidity. Animals were monitored daily and weighed weekly to ensure they were in good health. For tumor establishment, mice (n = 5) received an intrafemoral injection of HT1080 cells (5,000 cells in 20 μL of culture medium) under inhalation isoflurane anesthesia, with analgesia provided by subcutaneous carprofen (5 mg/kg). HT1080 cells (a widely used established human chondrosarcoma cell line) were engineered to express luciferase to enable longitudinal weekly monitoring of tumor growth by bioluminescence imaging (BLI) the tumor growth to be monitored weekly by bioluminescent imaging (BLI).^8^ For BLI, mice were injected intraperitoneally with 150 mg/kg D-luciferin (*in vivo* Glo, Promega) and anaesthetised with isoflurane prior to measurement of total flux (photons/s) using an IVIS Spectrum (PerkinElmer).

### *Ex Vivo* Imaging

After the final imaging session, the mice (n = 5) were euthanised by cervical dislocation. Both hindlimbs, along with tissue of interest were removed from each of the mice. The left and right hindlimbs were imaged from both the ventral and dorsal positions, under the same settings used to acquire the *in vivo* images. The hindlimbs were further dissected to separate the femur and proximal thigh muscle tissue from the lower portion of the hindlimb. The femurs and associated muscle tissue was then imaged using the same parameters as previously described. IVIS images were analyzed using Living Image software (v 4.5.5, PerkinElmer) by drawing ROIs around relevant tissues.

### *Ex Vivo* Biodistribution

Following *ex vivo* imaging, the femurs and surrounding muscle tissue, along with the other retained tissues were placed into preweighed counting tubes and weighed. The activity present in each sample was determined by measuring counts per minute (CPM) in a gamma counter. The CPM readings were then converted to units of activity (MBq) using a calibration curve generated from samples of known activities. Activity values were then decay-corrected to the time of administration of the immunoSCIFI probe and were normalized to the recorded injected activity values. The percentage of the injected activity per gram (%IA/g) for each tissue sample was calculated using the corrected activity value and sample mass.

### Statistical Analysis

GraphPad Prism v9.4.1 (GraphPad Software, San Diego, CA, USA) was used to create all graphical figures and perform all statistical analysis. One-tailed unpaired *t* tests were performed to compare probe uptake, fluorescence emissions, and femur-to-muscle ratios between involved and contralateral tissue. Data is reported as mean ± standard deviation, unless otherwise specified. Statistical significance is indicated by asterisks where ns = not significant, * = *p* < 0.05, ** = *p* < 0.01.

## Supplementary Material



## Notes

Absorbance/emission spectra of BOD665 dye, additional *in vivo* and *ex vivo* images (PDF)

## Abbreviations

Ab: antibody
*A*_max_: absorption maximum
ATCC: American Type Culture Collection
BLI: bioluminescence imaging
BODIPY: boron-dipyrromethene
BOD665: BODIPY 650/665-X-NHS ester
CO_2_: carbon dioxide
DMEM: Dulbecco’s modified Eagle medium
DFO: deferoxamine
DOL: degree-of-labeling
*E*_max_: emission maximum
IgG: immunoglobulin G
iTLC: instant thin-layer chromatography
IVIS: *in vivo* imaging system
MT1-MMP: membrane type 1 matrix metalloprotease
MWCO: molecular weight cut off
NHS: *N*-hydroxysuccinimide
NIR: near-infrared
NIRF: near-infrared fluorescence
NSG: NOD SCID gamma
PBS: phosphate buffered saline
PET: positron emission tomography
p.i.: postinjection
RCP: radiochemical purity
RIC: radioimmunoconjugate
rpm: revolutions per minute
RPMI: Roswell Park Memorial Institute Medium
SDS-PAGE: sodium dodecyl sulfate polyacrylamide gel electrophoresis
^89^Zr: zirconium-89
%IA/g: percentage injected activity per gram
