# Supplementary material for: In Vivo Evaluation of a Self-Excitatory Near-Infrared ImmunoSCIFI Probe
Source: Bioconjug Chem. 2026 Jan 7;37(1):93–9. doi: 10.1021/acs.bioconjchem.5c00506 (PMC12828711; doi:10.1021/acs.bioconjchem.5c00506)
Supplement: Supplementary file 1 [file bc5c00506_si_001.pdf]

Supplementary Information:

In Vivo Evaluation of a Self-Excitatory Near-Infrared ImmunoSCIFI Probe

Katie Gristwood[1], Saimir Luli[2], Helen J Blair[3,4], Kenneth S. Rankin[3,5], James C. Knight[1,6,*]

[1] School of Natural and Environmental Sciences, Newcastle University, Newcastle upon Tyne, NE1 7RU, UK.

[2] Preclinical In Vivo Imaging, Translational and Clinical Research Institute, Newcastle University, Newcastle upon Tyne, NE2 4HH, UK.

[3] Translational and Clinical Research Institute, Newcastle University, Newcastle upon Tyne, NE1 7RU, UK.

[4] Wolfson Childhood Cancer Research Centre, Newcastle Upon Tyne NE1 7RY, U.K.

[5] North of England Bone and Soft Tissue Tumour Service, Newcastle Upon Tyne Hospitals NHS Foundation Trust, Freeman Road, Newcastle Upon Tyne NE7 7DN, U.K.

[6] Newcastle Centre for Cancer, Newcastle University, Newcastle Upon Tyne NE1 7RU, U.K.

Supplementary Figure 1 – BOD665 absorption and SCIFI emission spectra

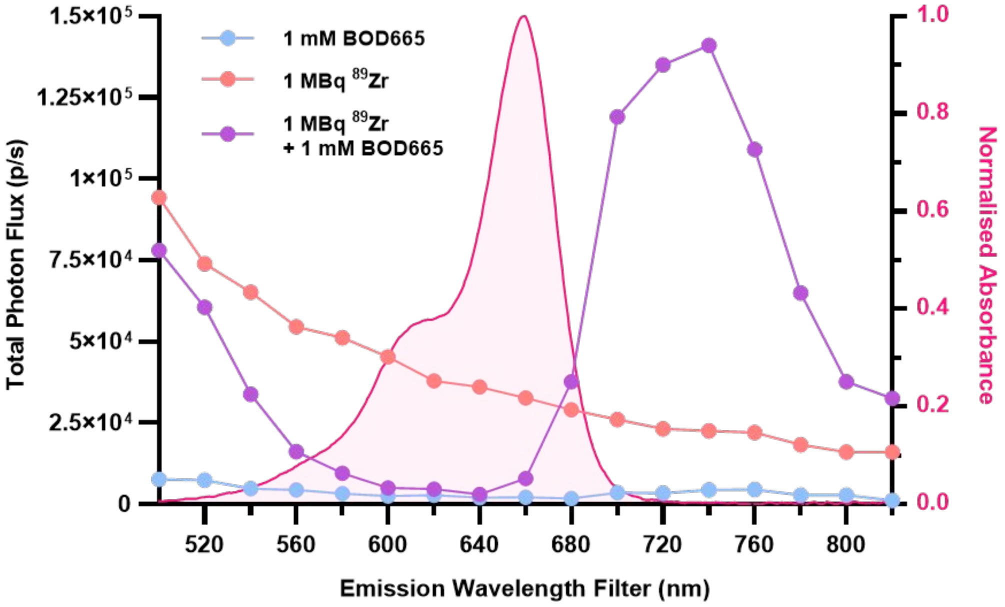

Figure S1. *Total photon (photons/second; p/s) flux values acquired on an IVIS scanner with emission wavelength filters ranging between 500-820 nm (20 nm bandwidth) from solutions of BOD655 (1 mM; blue), [^{89}Zr]Zr (1 MBq; orange) and BOD665 (1 mM) combined with [^{89}Zr]Zr (1 MBq) (purple). The absorbance of BOD665 is shown (pink). A reduction in photon flux between 520-680 nm observed in the combined solution of BOD665 and [^{89}Zr]Zr is due to the transfer of Cerenkov luminescence (CL) photon energy to the fluorophore.*

Supplementary Figure 2 – *UV–Vis absorbance spectra of unmodified anti-MT1-MMP and the anti-MT1-MMP-BOD665 immunoconjugates*

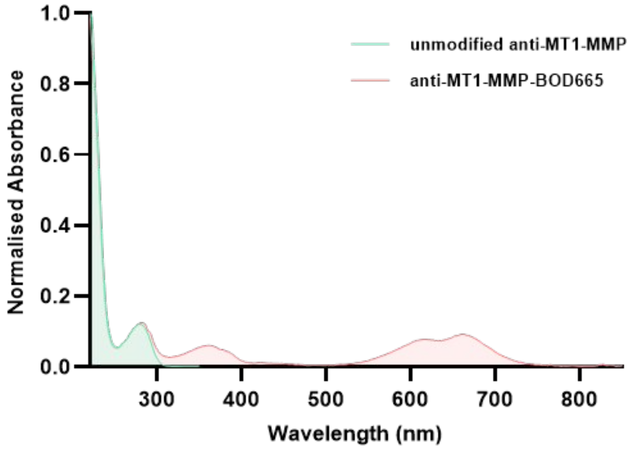

Figure S2. *Overlaid UV–Vis absorbance spectra of unmodified anti-MT1-MMP (green) and the anti-MT1-MMP-BOD665 immunoSCIFI conjugate (red).*

Supplementary Table 1 – Absorbance values and associated experimental parameters used to calculate the Degree-of-Labelling (DOL_{BOD665}) value for anti-MT1-MMP-BOD665

A_{280}	A_{665}	Volume (µL)	Correction factor *(A_{280}/A_{665} for BOD665)*	DOL_{BOD665}
3.79	1.81	52	0.12	2

Table S1. *Measured absorbance values, and associated parameters used in conjunction with the equations provided in the main article to calculate the DOL_{BOD665} value.*

Supplementary Figure 3 – *Radio-iTLC*

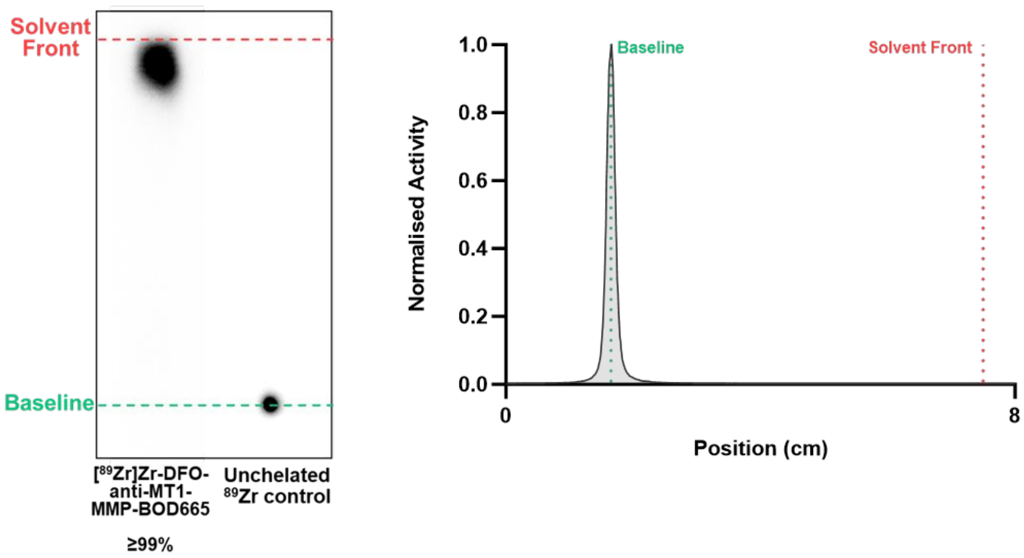

Figure S3. *Radiolabelling efficiency determination of [^{89}Zr]Zr-DFO-MT1-MMP-BOD665 compared to unchelated ^{89}Zr using radio-iTLC (left) and radio-iTLC analysis (right). The radiolabelling efficiency of the immunoSCIFI probe is expressed as a percentage.*

Supplementary Figure 3 – Additional *in vivo* IVIS images

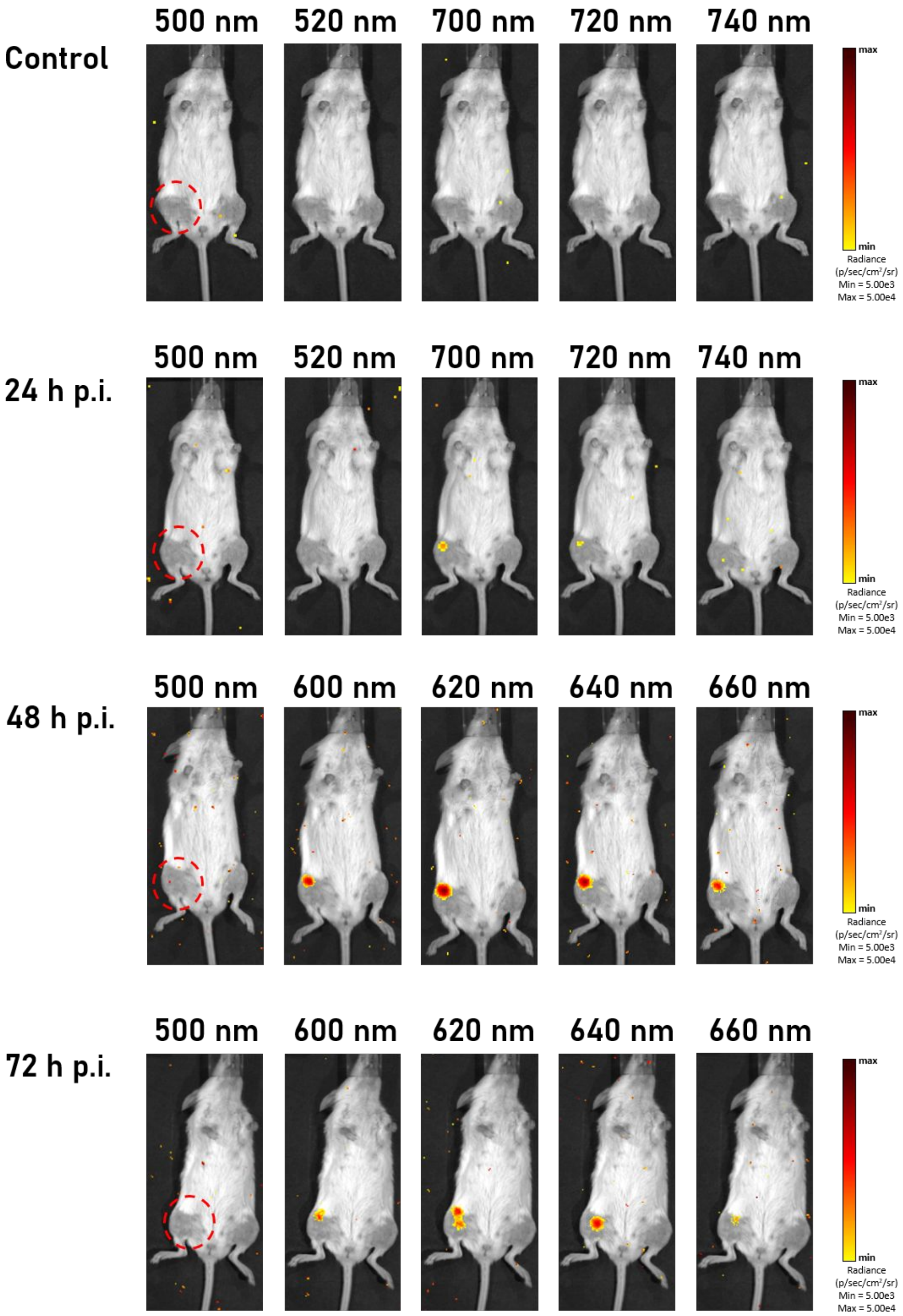

Figure S3. *Additional representative IVIS images showing a mouse bearing HT1080 (high MT1-MMP) sarcoma tumours (right femur in supine position; red dashed circle) acquired at 24, 48 and 72 h p.i. of [^{89}Zr]Zr-DFO-anti-MT1-MMP-BOD665. Control mice (representative image) bore femoral HT1080 tumours but did not receive the immunoSCIFI probe. Mice were imaged for Cerenkov luminescence (ex. block, em. 500–520 nm) and fluorescence/near-infrared fluorescence (ex. block, em. 600–740 nm).*

Supplementary Figure 4 – Muscle IVIS Images

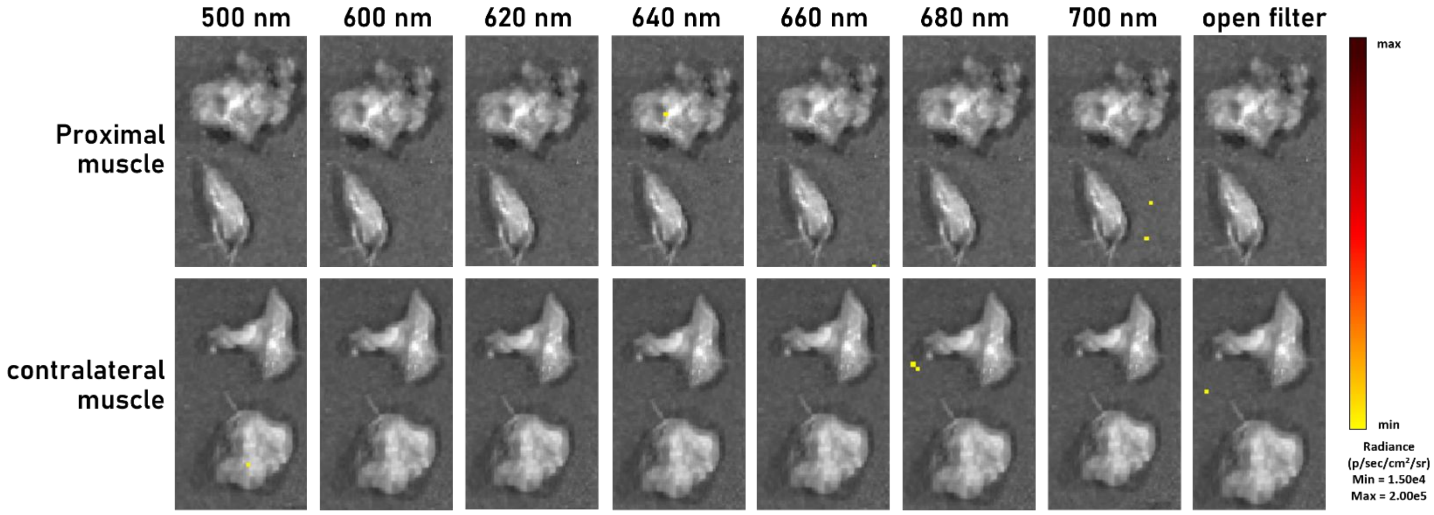

Figure S4. *Representative IVIS images showing ex vivo muscle tissue, proximal and contralateral to the femur bearing a HT1080 tumour. Tissue was imaged Cerenkov luminescence (ex. block, em. 500 nm) and SCIFI emissions (ex. block, em. 600–700 nm and open filter).*
